# rsEGFP2 enables fast RESOLFT nanoscopy of living cells

**DOI:** 10.7554/eLife.00248

**Published:** 2012-12-31

**Authors:** Tim Grotjohann, Ilaria Testa, Matthias Reuss, Tanja Brakemann, Christian Eggeling, Stefan W Hell, Stefan Jakobs

**Affiliations:** 1 Department of NanoBiophotonics, Max Planck Institute for Biophysical Chemistry, Göttingen, Germany; 2 Abberior Instruments GmbH, Göttingen, Germany; 3 Science for Life Laboratory, Advanced Light Microscopy, Karolinska Institutet, Solna, Sweden; 4 Department of Neurology, University of Göttingen, Göttingen, Germany; Harvard University, United States

**Keywords:** confocal microscopy, fluorescent probes, GFP, nanoscopy, superresolution, live-cell imaging, None

## Abstract

The super-resolution microscopy called RESOLFT relying on fluorophore switching between longlived states, stands out by its coordinate-targeted sequential sample interrogation using low light levels. While RESOLFT has been shown to discern nanostructures in living cells, the reversibly photoswitchable green fluorescent protein (rsEGFP) employed in these experiments was switched rather slowly and recording lasted tens of minutes. We now report on the generation of rsEGFP2 providing faster switching and the use of this protein to demonstrate 25–250 times faster recordings.

**DOI:** http://dx.doi.org/10.7554/eLife.00248.001

## eLife digest

For decades it was assumed that the diffraction of light meant that optical microscopy could not resolve features that were smaller than about the half the wavelength of the light being used to create an image. However, various ‘super-resolution’ methods have allowed researchers to overcome this diffraction limit for fluorescence imaging, which is the most popular form of microscopy used in the life sciences. This approach involves tagging the biomolecules of interest with fluorescent molecules, such as green fluorescent protein (GFP), so that they can be identified in cells. An excitation laser then drives the fluorescent molecule, which is also known as a fluorophore, into an excited state: after a short time, the fluorophore can return to its ground state by releasing a fluorescence photon. Images of the sample are built up by detecting these photons.

In STED super-resolution microscopy a second laser is used to instantly send the molecules from their excited or ‘on’ states back to their ground or ‘off’ states before any fluorescence can occur. The second laser beam is usually shaped like a doughnut, with a small region of low light intensity surrounded by a region of much higher intensity. STED microscopy is able to beat the diffraction limit because the second laser turns all the fluorophores ‘off’ except those in the small sub-wavelength region at the centre of the doughnut. The image is build up by scanning both lasers over the sample so that the small region in which the fluorophores are ‘on’ probes the entire cell.

RESOLFT is a similar technique that employs fluorescent molecules with ‘on’ and ‘off’ times that are much longer than those used in STED microscopy. In particular, RESOLFT uses fluorescent molecules that can be rapidly switched back and forth between long-lived ‘on’ and ‘off’ states many times by the two lasers. The fact that both these states are long-lived states means that RESOLFT requires much lower laser intensities than STED, which makes it attractive for imaging biological samples over large areas or long times.

RESOLFT demonstrated its suitability for bioimaging for the first time last year, with a protein called rsEGFP (reversibly switchable enhanced GFP) being employed as the fluorophore. However, the time needed to switch this protein between the ‘on state’ and the ‘off state’ was relatively long, and it took about an hour to record a typical image. Now, Grotjohann et al. have modified this protein to make a new fluorophore called rsEGFP2 with a shorter switching time, and have used it to image various structures—including Vimentin, a protein that forms part of the cytoskeleton in many cells, and organelles called peroxisomes—inside live mammalian cells. They were able to record these images some 25–250 times faster than would have been possible with previous RESOLFT approaches. The combination of RESOLFT and rsEGFP2 should allow researchers to image a wide variety of structures and processes in living cells that have not been imaged before.

**DOI:** http://dx.doi.org/10.7554/eLife.00248.002

## Introduction

While for many decades lens-based optical microscopy could not resolve features finer than half the wavelength of light (λ/2 > 200 nm), the recent development of fluorescence nanoscopy or super-resolution imaging methods boosted its resolution potential (Hell, 2003, 2009; Huang et al., 2010). In all these methods, the diffraction barrier is overcome by employing a fluorophore transition between two states, typically a fluorescent on- and a non-fluorescent off-state (Hell, 2009) in order to separate neighboring features. The method called RESOLFT differs from stochastic single fluorophore on-off-switching methods by the fact that a doughnut or a line pattern is scanned across the sample, determining at any point in time the nanosized coordinate range where the fluorophores are in the on-state. Using long lifetimes of the on- and off-states reduces the light intensities required for optical switching by orders of magnitude over the related STED approach, making RESOLFT attractive for extended live-cell or large area imaging.

Although it was suggested almost a decade ago (Hell, 2003; Hell et al., 2003) and its viability shown in principle (Hofmann et al., 2005; Dedecker et al., 2007; Schwentker et al., 2007) the wider applicability of RESOLFT nanoscopy to biological imaging has been demonstrated only recently (Brakemann et al., 2011; Grotjohann et al., 2011; Rego et al., 2012). The reason is that fluorescent protein based RESOLFT critically relies on reversibly switchable fluorescent proteins (RSFPs) providing many on-off cycles, such as the recently designed rsEGFP (Grotjohann et al., 2011). Unfortunately, in these demonstrations, the still relatively slow switching kinetics of rsEGFP entailed pixel dwell times of 10–20 ms at switching light intensities of ~1 kW/cm^2^. Hence images of 10 µm × 10 µm in size and a pixel step size of 20 nm required recording times of about an hour. In this study we describe the generation, characterization and application of a novel RSFP, namely rsEGFP2, which facilitates faster switching and, together with modifications of the initial switching scheme, enables 25–250 faster RESOLFT recordings in living cells.

## Results and discussion

**Figure 1.**
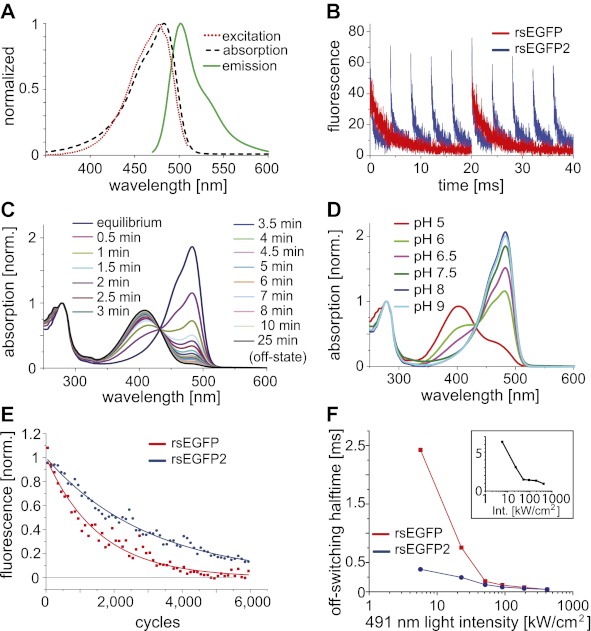
Characteristics of rsEGFP2. (**A**) Absorption (black dashed line), excitation (red dotted line), and emission (green solid line) spectra of rsEGFP2 in its equilibrium state at pH 7.5. (**B**) Switching curves of rsEGFP2 (blue) and rsEGFP (red). Switching was performed on purified proteins immobilized in a PAA-layer (pH ~6.5) by alternating irradiation with 491 nm (~2 kW/cm^²^) and 405 nm light (~2 kW/cm^²^, 40 µs). Fluorescence was recorded only during irradiation with light of 491 nm. Each curve is an average over 10 switching cycles. (**C**) Changes in the absorption spectrum of rsEGFP2 upon switching with light of 488 nm from the equilibrium to the off-state. The spectra were taken at the indicated time points and recorded on purified rsEGFP2 at pH 7.5 (**D**) Absorption spectra of equilibrium-state rsEGFP2 at different pH values. The absorption bands at 408 nm and 503 nm presumably correspond to the protonated and the de-protonated cis-chromophore, respectively. (**E**) Switching fatigue of rsEGFP2 (blue) and rsEGFP (red). Switching was performed on living PtK2 cells expressing Vimentin-rsEGFP or Vimentin-rsEGFP2 by alternate irradiation with 405 nm (2 kW/cm^²^) and 491 nm (5.7 kW/cm^²^) light. Illumination times were chosen so that the fluorescence was fully switched to the minimum or maximum, respectively, in each cycle. Each plotted data point is the average (over 100 cycles) of the maximum fluorescence intensity in each cycle. The data points were fitted by a mono-exponential function, and the resulting curve was baseline corrected and normalized to 1. (**F**) Comparison of the ensemble off-switching halftimes (defined as the time after which the fluorescence reached 50% of its initial value) of rsEGFP (red) and rsEGFP2 (blue) at different 491 nm light intensities. On-switching 405 nm light was kept constant (3 kW/cm^2^). Data were collected on living PtK2 cells expressing Vimentin-rsEGFP or Vimentin-rsEGFP2, respectively. Inset: Graph showing the ratio (R) of the off-switching halftime of rsEGFP divided by the off-switching halftime of rsEGFP2 against the 491 nm light intensity. **DOI:** http://dx.doi.org/10.7554/eLife.00248.003

We observed that already the exchange of a single amino acid in the chromophore of the photostable and widely-used enhanced green fluorescent protein (EGFP) (Patterson et al., 1997; Tsien, 1998), namely replacing threonine 65 by alanine, transformed EGFP into a fast switching RSFP, which exhibited a ‘negative switching mode’. ‘Negative switching’ RSFPs switch from the on- into the off-state at the wavelength, here ~480 nm, that is used to elicit fluorescence. The switching from the off-state into the on-state is achieved by irradiation with a different wavelength, here ~405 nm. Protein solutions of EGFP(T65A) exhibited a high residual fluorescence (~50% of the on-state signal) when the protein solution was switched into the off-state. However, analysis of EGFP(T65A) demonstrated fast switching and low switching fatigue, indicating that the different chromophore (an Ala-Tyr-Gly chromophore instead of a Thr-Tyr-Gly chromophore) might provide additional possibilities for the generation of fast switching RSFPs. Hence, we also introduced the mutation A206K to ensure that the protein is a true monomer (Zacharias et al., 2002) and introduced, alone or in combination, the four mutations that discriminate rsEGFP from EGFP. We screened this collection of EGFP variants for those exhibiting a high resistance against switching fatigue in combination with fast switching at light intensities previously used for RESOLFT (few kW/cm^2^). We found that introducing two mutations was sufficient to transform EGFP(T65A, A206K) into a novel RSFP with improved properties when compared to rsEGFP (Figure 1). We named this new variant (EGFP[T65A, Q69L, V163S, A206K]) (Figure 1—figure supplement 1) reversibly switchable EGFP2 (rsEGFP2). Note that rsEGFP has a TYG chromophore, whereas rsEGFP2 has an AYG chromophore.

**Figure 1—figure supplement 1.**
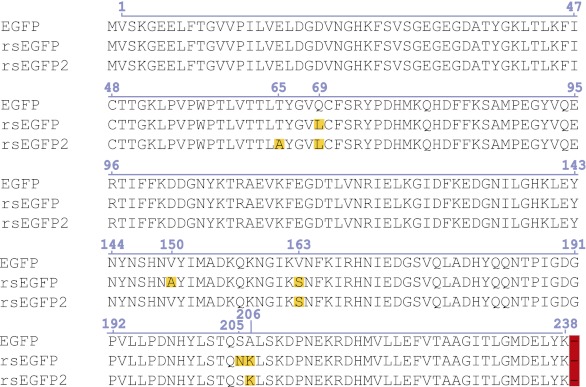
Alignment of the amino acid sequences of EGFP (GenBank Accession #U55762), rsEGFP (GenBank Accession #JQ969017), and rsEGFP2. Differences are highlighted. **DOI:** http://dx.doi.org/10.7554/eLife.00248.004

**Figure 1—figure supplement 2.**
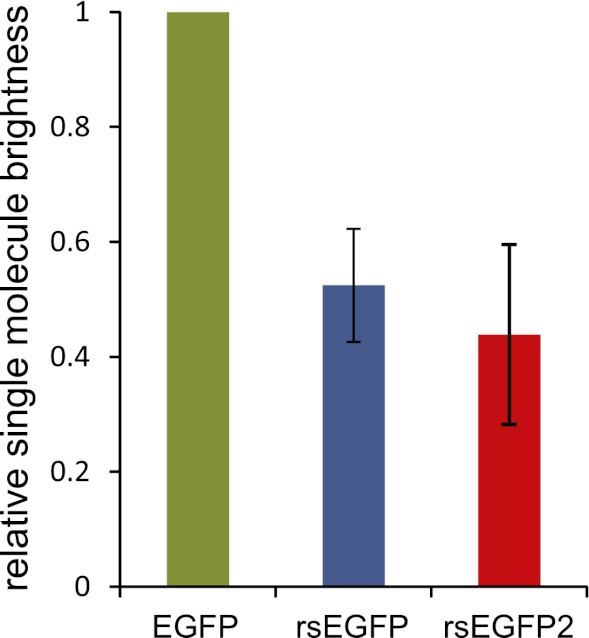
Single-molecule brightness values of EGFP, rsEGFP, and rsEGFP2 measured in PBS buffer (pH 7.5). Average and standard deviation of >30 FCS measurements at various laser intensities between 5 and 100 kW/cm^2^. Values normalized to EGFP. The error bars represent the error in the FCS experiments with the value for EGFP normalized to 1 in each individual experiment. **DOI:** http://dx.doi.org/10.7554/eLife.00248.005

**Figure 1—figure supplement 3.**
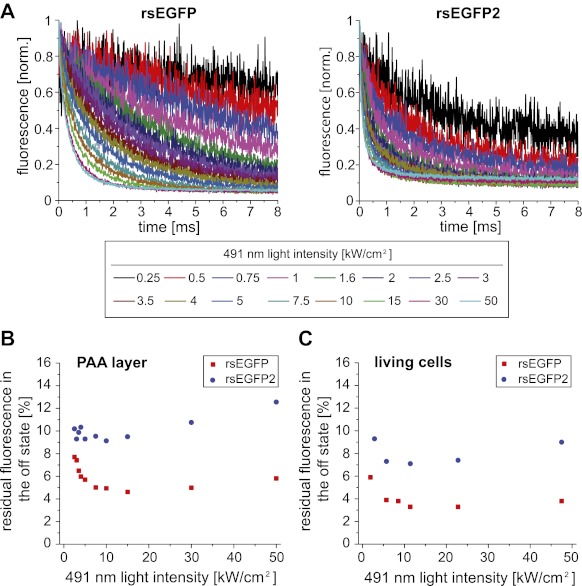
Off-switching speed of rsEGFP and rsEGFP2. (**A**) Off-switching kinetics of rsEGFP and rsEGFP2 embedded in a PAA layer (pH ~6.5) determined at different intensities of the 491 nm off-switching light. Each curve is an average of 100 measurements. In each measurement the proteins were switched into the on-state for 40 µs with 405 nm light (2 kW/cm^²^) and subsequently the decay of fluorescence was recorded over time at the indicated 491 nm light intensities. (**B**) Dependence of the residual fluorescence (off-state fluorescence) in the ensemble off-state as a function of the off-switching light intensity. (**C**) Dependence of the residual fluorescence (off-state fluorescence) in the off-state of Vimentin-rsEGFP or Vimentin-rsEGFP2 in living Ptk2 cells as a function of the off-switching light intensity. **DOI:** http://dx.doi.org/10.7554/eLife.00248.006

Adopting the fluorescent state at equilibrium, rsEGFP2 has its fluorescence excitation and emission maximum at 478 nm and 503 nm, respectively (Figure 1A). In the fluorescent state, a solution of rsEGFP2 exhibits an extinction coefficient of *ε* ≈ 61,000 M^−1^cm^−1^ and a fluorescence quantum yield of *Φ*_*Fl*_ = 0.3 (Table 1). Hence the brightness (given by *ε Φ*_*Fl*_) of rsEGFP2 is similar to that of rsEGFP and about 60% of that of EGFP at low light intensity irradiation. Single-molecule spectroscopy measurements performed at higher light intensities (5 to 100 kW/cm^2^) showed that the single molecule brightness of rsEGFP and rsEGFP2 were ~52% and ~44% of that of EGFP, respectively (Figure 1—figure supplement 2). The slight difference between brightness values determined at low and high light intensities may be attributed to the increased population of unidentified dark states at higher intensities. rsEGFP2 exhibits a ‘negative’ switching mode, that is irradiation with light of around 480 nm induces fluorescence and, in a competing process, switches rsEGFP2 off. Subsequent irradiation with light of around 405 nm switches the protein back from the off- into the on-state (Figure 1B). Irradiation of a purified rsEGFP2 solution with light of ~480 nm leads to a decrease of the 483 nm absorption band, which presumably corresponds to the anionic (de-protonated) cis-chromophore, and to the onset of an absorption band peaking at 408 nm, presumably corresponding to the neutral (protonated) trans-chromophore state (Andresen et al., 2005; Andresen et al., 2007) (Figure 1C). The single molecule brightness, that is the number of emitted photons per time unit, of rsEGFP and rsEGFP2 are similar. However, because a single switching cycle of rsEGFP2 is on average shorter than a single switching cycle of rsEGFP, rsEGFP2 emits fewer photons in a single cycle than rsEGFP. The p*K*_a_ of the chromophore in the thermal equilibrium state is ~5.8 (Figure 1D). Hence the p*K*_a_ of rsEGFP2 is lower by 0.7 pH units than the p*K*_a_ of rsEGFP. As reported previously for similar RSFPs, the light driven switching of rsEGFP2 is likely due to a cis/trans isomerization of the chromophore, accompanied by a change of the chromophoric protonation state (Andresen et al., 2005; Andresen et al., 2007; Bourgeois and Adam, 2012).

**Table 1.** Comparison of EGFP, rsEGFP and rsEGFP2 properties. **DOI:** http://dx.doi.org/10.7554/eLife.00248.007

	EGFP	rsEGFP	rsEGFP2
Extinction coefficient (ε) [M^−1^ cm^−1^]	53.000 *	47.000	61.300
Fluorescence quantum yield (Ф^FL^)	0.6*	0.36	0.3
Excitation maximum on-state [nm]	489	493	478
Emission maximum [nm]	509	510	503
Absorption maximum off-state [nm]	n.a.	396	408
Number of switching cycles to bleach to 50% of the initial fluor in the on-state (2 kW/cm^2^ at 405 nm, 5.7 kW/cm^2^ at 491 nm)‡	n.a.	∼1,100	∼2,100
Chromophore maturation halftime at 37°C [min]	∼25†	∼180	∼20

* Patterson et al., 1997.

† Verkhusha et al., 2001.

‡ Note, that the number of cycles may depend on the sample preparation and the experimental conditions.

n.a.: not applicable.

The total number of switching cycles before bleaching is critical for the usability of a RSFP for RESOLFT microscopy (Hell, 2009). To compare rsEGFP2 with rsEGFP in this regard, we immobilized purified proteins in polyacrylamide layers (PAA). Applying light intensities that have previously been used for RESOLFT (few kW/cm^2^), we recorded the fluorescence during 6,000 on-off switching cycles (Figure 1E). The illumination times were adapted for the two proteins such that the signal reached a maximum or minimum in each cycle. We found that under these conditions the fluorescence was halved not before ~1,100 (rsEGFP) and ~2,100 (rsEGFP2) cycles, demonstrating that rsEGFP2 accommodates even more switches than rsEGFP.

In most negative switching RSFPs, including rsEGFP, the ensemble off-switching with blue light is slower by 2–3 orders of magnitude, compared to the on-switching with UV light at comparable light intensities, rendering the off-switching the time-limiting step in RESOLFT microscopy. We compared the off-switching half-time (time after which the fluorescence signal is reduced to 50%) of rsEGFP2 and rsEGFP as a function of the off-switching light intensity (491 nm) (Figure 1F). We found that the difference in the off-switching halftimes of rsEGFP2 and rsEGFP depends on the light intensities applied. The lesser the light intensities, the more pronounced is the speed advantage of rsEGFP2. At a light intensity of 5.5 kW/cm^2^ the off-switching of rsEGFP2 was ~6.5 times faster than the off-switching of rsEGFP (Figure 1F, inset). Presumably the switching speed advantage for rsEGFP2 is even higher at lower intensities. For undesirable intensities of >100 kW/cm^2^, the differences between rsEGFP and rsEGFP2 are negligible (Figure 1F, inset). These measurements also revealed that the level to which the fluorescence intensity of an ensemble of rsEGFP2 proteins can be reduced (the off-state fluorescence), depends on the intensities for off-switching (Figure 1—figure supplement 3). Hence the light intensities and the irradiation times used for off-switching influence both the switching speed as well as the lowest residual fluorescence. Furthermore, we observed that the buffer conditions, most notably the pH, and the cellular environments also influence the absolute switching speed, potentially requiring adaptations of the illumination protocol to the observed samples.

**Figure 2.**
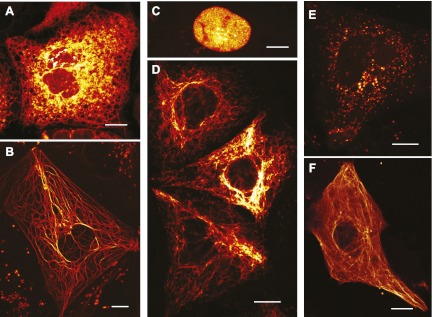
Expression of various functional rsEGFP2 fusion proteins in mammalian cells. (**A**) rsEGFP2-KDEL (targeting to the ER), (**B**) Keratin19-rsEGFP2, (**C**) Histone H2B-rsEGFP2, (**D**) Vimentin-rsEGFP2, (**E**) Pex16-rsEGFP2, and (**F**) rsEGFP2-alpha-tubulin. Shown are single confocal sections (**C**, **F**) and maximum intensity projections of confocal images (**A**, **B**, **D**, **E**) recorded on living cells. Fluorescence was excited by simultaneous irradiation with light of 488 nm and 405 nm. (**A**–**E**): PtK2 cells; (**F**): Vero cell. Scale bars: 10 μm. **DOI:** http://dx.doi.org/10.7554/eLife.00248.008

In mammalian cells, rsEGFP2 can be fused to histone H2B and alpha-tubulin, which require a truly monomeric fusion tag (Figure 2). Corroborating its monomeric nature, it migrates on semi-native gels in a single band of the expected size (Figure 2—figure supplement 1). rsEGFP2 maturates in vitro at 37°C with a halftime of ~20 min, which is shorter or comparable to most conventional fluorescent proteins (Shaner et al., 2008) including mCherry (15 min), tdTomato (60 min) or TagRFP (100 min). Its maturation time is superior to both rsEGFP (3 hr) (Grotjohann et al., 2011) and a maturation-improved Dronpa(M159T) variant (Stiel et al., 2007; Willig et al., 2011) (~50 min). Presumably because of its fast maturation time and due to the fact that its linker is identical to that of EGFP (which has been proven to be very suitable for the generation of fusion proteins) rsEGFP2 is well suited to tag proteins in living mammalian cells (Figure 2).

**Figure 2—figure supplement 1.**
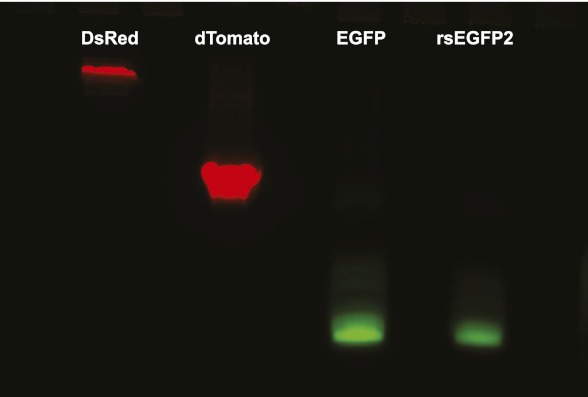
Semi-native polyacrylamide gel electrophoresis of rsEGFP2. Purified monomeric EGFP, dimeric dTomato, tetrameric DsRed, and rsEGFP2 were separated on a semi-native gel (a two-phase polyacrylamide gel) consisting out of a 12.5% separation gel (6.3 ml H_2_O, 5 ml 1.5 M Tris–HCl pH 8.8, 8.3 ml Rotiphorese Gel 30 solution [Roth, Karlsruhe, Germany], 200 μl 10% [wt/vol] sodiumdodecyl sulphate [SDS], 200 μl 10% [wt/vol] ammonium persulfate (APS), 20 μl Tetramethylethylendiamin [TEMED]) and a 5% loading gel (5.6 ml H_2_O, 2.5ml 1.5 M Tris–HCl pH 6.8, 1.7 ml Rotiphorese Gel 30 solution, 100 μl 10% [wt/vol] SDS, 100 μl 10% [wt/vol] APS, 10 μl TEMED). Images were taken with a custom-built gel documentation system. To detect green fluorescence (EGFP and rsEGFP2) the gel was irradiated with light of 470 ± 5 nm and fluorescence was detected at 525 ± 30 nm. To detect red fluorescence (dTomato and DsRed) the gel was irradiated with light of 545 ± 10 nm and fluorescence was recorded at 617 ± 37. Both images were overlaid and are represented in false colors. **DOI:** http://dx.doi.org/10.7554/eLife.00248.009

**Figure 3.**
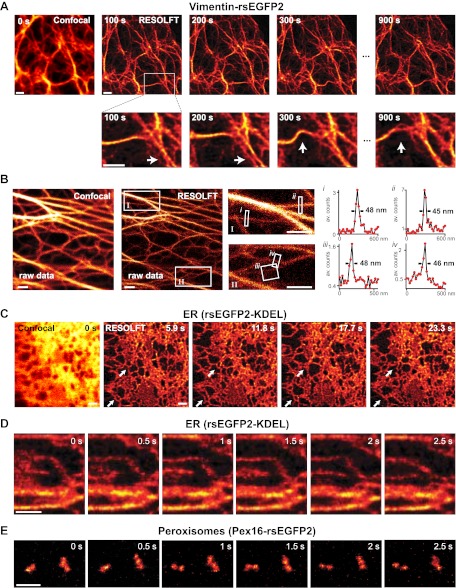
RESOLFT time lapse imaging using rsEGFP2 in living mammalian PtK2 cells. (**A**) Cells expressing Vimentin-rsEGFP2: initial confocal (left) and subsequent RESOLFT images taken every 100 s. Lower row: magnifications of the indicated areas. (**B**) Lateral resolution measurement: raw images of cells expressing Keratin19-rsEGFP2 recorded with a RESOLFT Quad P microscope (Abberior Instruments GmbH, Göttingen, Germany) with similar imaging conditions as in (**A**) (on: 405 nm, 5 kW/cm^²^, 20 µs; off: 488 nm, 34 kW/cm^²^, 360 µs; read-out: 488 nm, 76 kW/cm^²^, 20 µs). From left to right: confocal raw image and corresponding raw RESOLFT image. Magnifications of the boxed areas in the RESOLFT image. The graphs show averaged line profiles across the indicated filaments (*i*–*iv*) within the respective boxes. The line profiles used for averaging were taken equidistant (20 nm) along the whole respective indicated area. (**C**),(**D**) rsEGFP2 targeted to the ER (rsEGFP2-KDEL): (**C**) 10 µm × 10 µm initial confocal (left) and subsequent RESOLFT images recorded every 5.9 s, and (**D**) 2.8 µm × 3.2 µm RESOLFT image-series imaged at 2 Hz. (**E**) RESOLFT imaging of peroxisomes labeled by Pex16-rsEGFP2 fusion proteins. Pixel step sizes: 20 nm (**A**, **B**) and 40 nm (**C**–**E**). Pixel dwell times: 380 µs (**A**), 400 µs (**B**), 75 µs (**C**, **D**), and 120 µs (**E**). In (**D**) and (**E**) pixels were interpolated to a size of 20 nm × 20 nm. The arrows indicate moving structures. Richardson Lucy restoration was used for all RESOLFT images except (**B**). Scale bars: 1 µm. **DOI:** http://dx.doi.org/10.7554/eLife.00248.010

The possibility to switch rsEGFP2 faster than rsEGFP, its higher switching stamina, and its faster maturation kinetics, suggested that this protein would be superior for fast live-cell RESOLFT imaging. We first expressed rsEGFP2 fused to the C-terminus of Vimentin in mammalian PtK2 (*Potorous tridactylis*) cells. RESOLFT imaging was performed with a home-built setup maintaining the cells at 35°C. Because the filaments formed by Vimentin-rsEGFP2 are relatively immobile, we selected an irradiation scheme encompassing low switching light intensities in combination with extended irradiation times. The living cells were imaged pixel-by-pixel, by first irradiating with 405 nm light (2 kW/cm^²^) for 40 µs to switch most proteins into their fluorescent on-state. After a short illumination break of 10 µs, the doughnut shaped 491 nm beam (10 kW/cm^²^, 300 µs) was used to switch rsEGFP2 into the off-state, confining the on-state to the doughnut center. Finally, the rsEGFP2 fluorescence was probed for 30 µs by irradiation with 491 nm light (38 kW/cm^²^). Hence the pixel dwell time was 380 µs, which is 25- to 50-fold faster than previously reported using rsEGFP on similar structures (Grotjohann et al., 2011). To enhance the image contrast, we also employed Richardson-Lucy restoration (Richardson, 1972; Hofmann et al., 2005). Movements of Vimentin-filaments in a living cell were recorded in 10 µm × 10 µm frames of 20 nm pixel size, every 100 s (Figure 3a; Figure 3—figure supplement 1; Movie 1). After the 20th image, the overall fluorescence still amounted to 65% of its initial value, underscoring the bleaching resistance of rsEGFP2.

**Figure 3—figure supplement 1.**
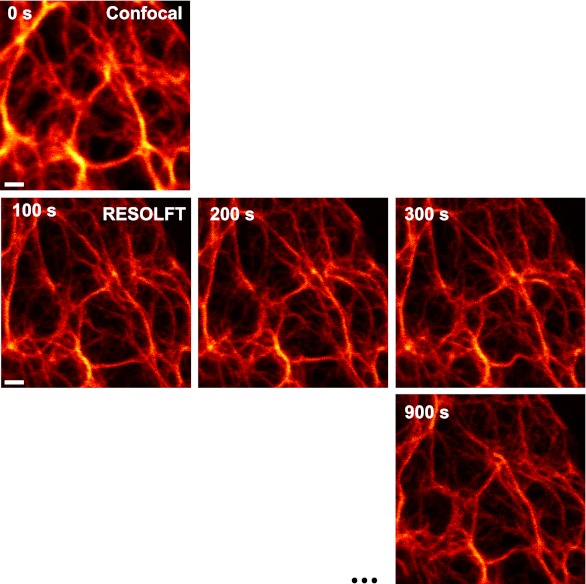
Raw RESOLFT images of Figure 3A. No image processing was applied. Shown are PtK2 cells expressing Vimentin-rsEGFP2: initial confocal (left) and subsequent RESOLFT images taken every 100 s. Scale bar: 1 µm. **DOI:** http://dx.doi.org/10.7554/eLife.00248.011

**Figure 3—figure supplement 2.**
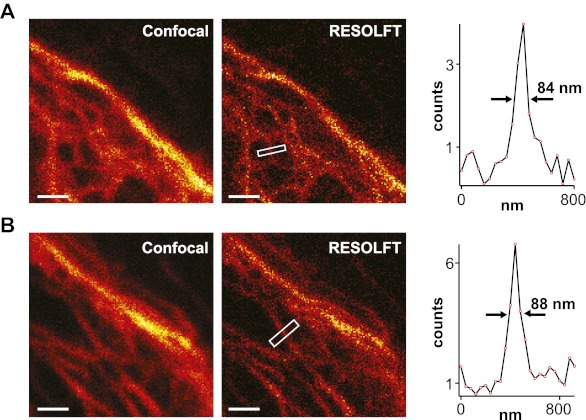
Lateral resolution in fast RESOLFT imaging. (**A**),(**B**) Typical examples. Shown are raw images of cells expressing Keratin19-rsEGFP2 taken on a RESOLFT Quad P microscope (Abberior Instruments GmbH, Göttingen, Germany) with a pixel dwell time similar as in Figure 3D,E. Imaging conditions: (on: 405 nm, 8 kW/cm^²^, 2 µs; off: 488 nm, 68 kW/cm^²^, 61 µs; read-out: 488 nm, 200 kW/cm^²^, 7 µs; pixel size: 40 × 40 nm). From left to right: confocal raw image and corresponding raw RESOLFT image. The graphs show averaged line profiles across the indicated filaments within the respective boxes. The line profiles used for averaging were taken equidistant (40 nm) along the whole respective indicated area. Scale bar: 1 µm. **DOI:** http://dx.doi.org/10.7554/eLife.00248.012

**Figure 3—figure supplement 3.**
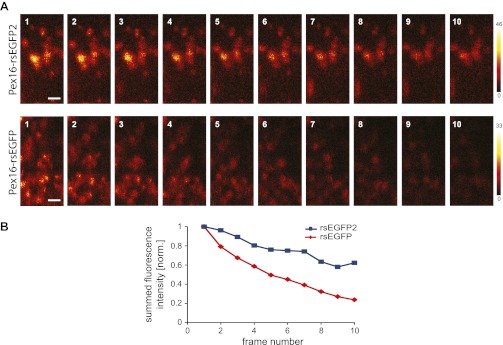
Comparison of rsEGFP and rsEGFP2 at RESOLFT imaging conditions. (**A**) Repeated imaging of peroxisomes labeled by Pex16-rsEGFP or Pex16-EGFP2 fusion proteins. Imaging conditions were as in Figure 3D. Pixel step size: 40 nm; on: 405 nm, 4 kW/cm^²^, 20 µs; off: 491 nm, 20 kW/cm^²^, 50 µs; read-out: 491 nm, 76 kW/cm^²^, 5 µs; pixel dwell time: 75 µs. Images were taken every 5 s. Shown are raw data. (**B**) Decay of the summed fluorescence intensities of the images shown in (**A**). Note that rsEGFP photobleaches faster than rsEGFP2. Scale bar: 1 µm. **DOI:** http://dx.doi.org/10.7554/eLife.00248.013

**Movie 1.** Animated sequence of RESOLFT recordings of a living PtK2 cell expressing Vimentin-rsEGFP2 as shown in Figure 3A. 20 RESOLFT images were taken every 100 s. Image size: 10 µm × 10 µm. The movie is accelerated by a factor of 200 compared to the original recording speed. **DOI:** http://dx.doi.org/10.7554/eLife.00248.014

To determine the obtainable resolution, we imaged cells expressing Keratin19-rsEGFP2 filaments using similar illumination conditions as before. Evaluating the intensity profiles of these filaments in the focal plane revealed a resolution <50 nm in raw image data (Figure 3B).

Next, we targeted rsEGFP2 to the lumen of the endoplasmic reticulum (ER). Since the ER is a fast moving structure we increased the recording speed further by doubling the light intensities (on: 405 nm, 4 kW/cm^²^, 20 µs; off: 491 nm, 20 kW/cm^²^, 50 µs; read-out: 491 nm, 76 kW/cm^²^, 5 µs) thus cutting the pixel dwell time down to 75 µs, which is ~250 times faster than reported previously using rsEGFP. We did not introduce an irradiation break within a single switching cycle. Because we aimed at capturing fast movements, we also increased the pixel size to 40 nm × 40 nm, thus covering a field of 100 µm² in 5.9 s (Figure 3C). Repeated imaging revealed fast changes occurring in the range of seconds in the highly interconnected ER. To visualize movements of the ER in the sub-second range, we reduced the field of view to ~9 µm^2^ corresponding to 0.5 s frame times. Figure 3D shows six RESOLFT images out of a time lapse movie containing 100 images (Movie 2) revealing changes in the ER structure occurring in <1 s. Since the ER-tubules are too large to determine the obtained resolution, we imaged again Keratin19-rsEGFP2 expressing cells using a similar dwell time and established <90 nm in raw data (Figure 3—figure supplement 2), meaning that speed was obtained at the expense of resolution.

**Movie 2.** Animated sequence of RESOLFT recordings of a living PtK2 cell expressing rsEGFP2 targeted to the ER as shown in Figure 3D. 100 RESOLFT images were taken every 0.5 s. Image size: 2.8 µm × 3.2 µm. The speed of the movie corresponds to the imaging speed. **DOI:** http://dx.doi.org/10.7554/eLife.00248.015

Likewise, when rsEGFP2 was fused to the protein Pex16 to highlight the peroxisomes in living PtK2 cells, we could follow the movement of individual peroxisomes in the RESOLFT-mode in a 3 µm × 2 µm field of view at 2 Hz (Figure 3E; Movie 3). Since the fluorescent proteins are confined to individual peroxisomes and do not diffuse over large distances, labeled peroxisomes are well suited for a direct comparison of rsEGFP2 and rsEGFP bleaching. The comparison showed that the overall photobleaching of rsEGFP2 was substantially lower than that of rsEGFP when the above fast RESOLFT imaging conditions were applied (Figure 3—figure supplement 3).

**Movie 3.** Animated sequence of RESOLFT recordings of a living PtK2 cell expressing Pex16-rsEGFP2 to highlight the peroxisomes as shown in Figure 3E. 20 RESOLFT images were taken every 0.5 s. Image size: 3 µm × 2 µm. The speed of the movie corresponds to the imaging speed. **DOI:** http://dx.doi.org/10.7554/eLife.00248.016

Phototoxicity is a major concern in (superresolution) fluorescence microscopy of living cells. Among the established methods, STED-microscopy entails relatively large intensities, (5–200 MW/cm^2^), depending on the desired resolution. However, since the wavelengths (560–800 nm) used for STED are in the comparatively benign long-wavelength part of the spectrum (Hell, 2009), STED microscopy can image living cells and tissues, including neurons in the cerebral cortex of a living mouse (Berning et al., 2012). In the RSFP-based RESOLFT microscopy demonstrated here, the employed light intensities (1–80 kW/cm^2^) are several orders of magnitude lower than in STED-microscopy and comparable to those used in live-cell confocal fluorescence microscopy. Stochastic single-molecule based approaches, such as the methods called PALM (Manley et al., 2008; Shroff et al., 2008), STORM (Jones et al., 2011; Shim et al., 2012), and GSDIM (Fölling et al., 2008), typically use similar light intensities for imaging living cells (0.1–100 kW/cm^²^ depending on the exposure time or camera frame rate), but they apply these intensities (i.e. temporal and spatial photon densities) continuously to all points in the imaged area. Since our RESOLFT approach has been implemented as a point-scanning system, the intensities employed are applied only for a brief duration on a small, sub-micrometer sized region of the imaged area. Hence any pixel is only illuminated during a small fraction of the recording time of the image. In the stochastic methods, the whole imaged area is irradiated for the entire time of recording, that is for a couple of seconds or minutes. Therefore, in the RESOLFT microscopy demonstrated here, the total light dose impinging on the cell is lower by 3–4 orders of magnitude compared to the stochastic single-molecule based approaches. Concretely, for recording the shown RESOLFT images, 2–10 J/cm^²^ were applied for switching into the on-state, and 25–300 J/cm^²^ for eliciting fluorescence and switching the protein off. PALM live-cell imaging reportedly requires light doses of 1,000–100,000 J/cm^²^ for on-switching with UV (405 nm) light and 25,000–300,000 J/cm^²^ for fluorescence excitation (Manley et al., 2008; Shroff et al., 2008). Live-cell GSDIM experiments using a yellow fluorescent protein required even larger irradiation doses (100,000–900,000 J/cm^²^) (Fölling et al., 2008; Testa et al., 2010). Although RESOLFT microscopy may induce phototoxicity after extended exposure, similarly to live-cell confocal microscopy, it currently is the superresolution method entailing the lowest light dose. Therefore, perhaps not surprisingly, RESOLFT microscopy has been used to image neurons in living organotypical hippocampal cultures over several hours without noticeable photodegradation (Testa et al., 2012). Note that the comparatively low-dose/low-intensity requirement of the RESOLFT concept is due to the fact that it uses long-lived on- and off-states in combination with the fact that it does not require fast emission of many photons for establishing molecular coordinates (localization). Since the applied intensities are largely determined by the on-off switching kinetics, the concept offers ample room for accommodating novel proteins with switching kinetics entailing even lower light doses and intensities.

In conclusion, rsEGFP2 is a bright, monomeric, photostable, quickly maturating, and fast switching alternative to rsEGFP with comparatively low photobleaching. We expect it to outperform other green fluorescent RSFPs (Ando et al., 2007; Stiel et al., 2007) because of its faster maturation and good usability for functional protein tagging. An implementation of quick illumination sequences allowed us to realize up to 250-fold faster recordings as compared to previous reports, thus facilitating live-cell RESOLFT nanoscopy with pixel dwell times down to 70 µs. Since one can adjust both the duration and the illumination intensity, as well as the optical switching scheme, RESOLFT nanoscopy allows one to adapt speed and resolution within a certain range, to the sample needs.

Finally, we note that in the point-scanning scheme used here, the total recording time of the image scales with the area of recording. Parallelization of the scanning procedure with an array of doughnuts or lines (so-called ‘structured illumination’) (Gustafsson, 2005; Schwentker et al., 2007; Rego et al., 2012) overcomes this dependence on the field of view and cuts down the recording time by the degree of parallelization. Owing to the low-light level operation, the degree of parallelization can easily amount up to a factor of 100–1000. For this reason, given the short pixel dwell times attained herein, parallelized RESOLFT versions should enable video-rate nanoscopy across the whole field of view of the objective lens.

## Materials and methods

### Mutagenesis

For site-directed mutagenesis, the QuikChange Site Directed Mutagenesis Kit (Stratagene, La Jolla, CA) or a multiple-site mutagenesis approach using several primers were used.

### Protein expression, purification and characterization

The experimental procedures were essentially as described previously (Grotjohann et al., 2011). In brief, proteins were expressed in the *E. coli* strain BL21-CP-RIL and purified by Ni-NTA affinity chromatography (His SpinTrap, GE Healthcare), according to the manufacturer's instructions. The purified proteins were concentrated by ultrafiltration and taken up in 100 mM Tris–HCl, 150 mM NaCl, pH 7.5.

For the determination of the absorption, excitation and emission spectra of rsEGFP2, a protein solution (pH 7.5) was analyzed with a Varian Cary 4000 UV/VIS photospectrometer and a Varian Cary Eclipse fluorescence spectrometer, respectively. At this pH, the majority of the equilibrium-state rsEGFP2 chromophores are in the deprotonated cis-state (see Figure 1D). To determine its emission spectrum, rsEGFP2 was excited at 460 nm; the excitation spectrum was determined by measuring fluorescence at 520 nm. The fluorescence quantum yields and the molar extinction coefficients at the respective absorption maximum were determined relative to the reported values of EGFP (quantum yield Φ_FL_ = 0.60, molar extinction coefficient at 489 nm *ε* = 53,000 M^−1^ cm^−1^) (Patterson et al., 1997). Irradiation-dependent changes in the absorption were quantified by illuminating the protein solution in a cuvette with a fiber coupled mercury lamp (Lecia Microsystems, Wetzlar, Germany) equipped with a (488 ± 5) nm excitation filter. For each measurement of the spectrum the irradiation was briefly interrupted.

For the embedding of rsEGFP2 in a PAA layer, 24.5 µl of purified rsEGFP2 (~0.1 mM) was mixed with 17.5 µl Tris–HCl pH 7.5, 30 µl acrylamide (Rotiphorese Gel 30, Roth, Karlsruhe, Germany), 0.75 µl 10 % ammonium persulfate and 1µl 10 % TEMED. About 10 µl of this solution was placed on a glass slide and a cover slip was pressed onto the sample. After complete polymerization, the sample was sealed with silicon-based glue (Picodent twinsil, Picodent, Wipperfürth, Germany).

### Determination of chromophore maturation halftime

To determine the time required for chromophore maturation in rsEGFP2, the *E. coli* cell strain TOP10 (Invitrogen, Carlsbad, CA) was transformed with the inducible expression plasmid pBad-rsEGFP2 and grown overnight at 37°C in LB-Amp medium. The overnight culture was used to inoculate 200 ml LB-Amp growth medium. At an OD600 of 0.5 to 0.6, addition of arabinose to a final concentration of 0.2% induced the protein expression. The cultures were further incubated at 37°C for 2 hr. Cells were opened up by several freeze–thaw cycles and pelleted by centrifugation. rsEGFP2 was purified immediately from the supernatant using a His SpinTrap column (GE Healthcare, Freiburg, Germany). The proteins were diluted in buffer (final concentration: 20 mM NaH_2_PO_4_, 500 mM NaCl, 30 mM imidazol, pH 7.5). Care was taken that all preparation steps took place at 4°C. Finally, fluorescence emission spectra of rsEGFP2 were taken at several time points using a fluorescence spectrometer (Varian Cary Eclipse) while incubating the protein solution at 37°C.

### Mammalian cell culture

PtK2 (*Potorous tridactylis*) cells were cultured under constant conditions at 37°C and 5% CO_2_ in DMEM (Invitrogen, Carlsbad, CA) containing 5% FCS (PAA, Pasching, Austria), 100 units per ml streptomycin, 100 µg/ml penicillin (all Biochrom, Berlin, Germany), and 1 mM pyruvate (Sigma, St. Louis, USA ). For transfection, cells were seeded on cover glasses in 6-well plates. At the next day, cells were transfected with plasmid DNA using Nanofectin (PAA, Pasching, Austria) according to the manufacturer's instructions. After 24 hr the growth medium was replaced. Cells were imaged 24–72 hr after transfection.

### Cloning

To generate the various fusion constructs of rsEGFP with Keratin19, with the histone H2B, with Vimentin, or with the peroxisomal membrane protein Pex16, rsEGFP was amplified (forward primer: GATCCACCGGTCGCGGCGTGAGCAAGGGCGAGGAGCTG/reverse primer: ACAACTTAAGAACAACAATTGTTACTTGTACAGCTCGTCCATGCC). The PCR fragment was cloned into the gateway destination vector pMD-tdEosFP-N using the restriction sites Age*I* and Afl*II*, thereby replacing the tdEosFP coding sequence against the rsEGFP2 sequence. The final plasmids pMD-Ker19-rsEGFP2, pMD-H2B-rsEGFP2, pMD-Vim-rsEGFP2 and pMD-Pex16-rsEGFP2 were constructed by gateway vector conversion (Invitrogen, Carlsbad, CA) using the donor vectors pDONR223-Krt19, pDONR223-Hist1H2BN, pDONR223-Vim and pDONR223-Pex16, respectively (Lamesch et al., 2007). Pex16-rsEGFP was cloned accordingly.

To generate pMD-rsEGFP2-α-Tubulin, rsEGFP2 was amplified (forward primer: GATCCGCTAGCGCTAATGGTGAGCAAGGGCGAGGAG/reverse primer: CACTCGAGATCTGAGTCCGGACTTGTACAGCTCGTCCATGCC) and cloned into the vector pEGFP-Tub (Clontech, Mountain View, CA) using the restriction sites Nhe*I* and Bgl*II* replacing EGFP.

To generate a construct that targets rsEGFP2 to the ER, the rsEGFP2 sequence was PCR-amplified (forward primer: CTGCAGGTCGACATGGTGAGCAAGGGCGAGGA/reverse primer: TTCTG CGGCCGCCTTGTACAGCTCGTCCATGCCGCCGGT). The PCR product was ligated into the vector pEF/myc/ER (Invitrogen, Carlsbad, CA) using the Sal*I* and Not*I* restriction sites.

### RESOLFT microscope

A home-built RESOLFT microscope (Grotjohann et al., 2011; Testa et al., 2012) was adapted for imaging rsEGFP2 in living cells. The microscope utilized three separate beam paths for generating focal spots: two at 491 nm wavelength for excitation and off-switching and one at 405 nm for on-switching of the fluorophores. The two focal spots at 491 nm comprised: (i) a normal diffraction-limited focus with a Gaussian profile for reading out the fluorescence signal and (ii) a focus with a central intensity minimum (‘zero’) for off-switching at the focal periphery in the xy-plane, obtained by passing the beam through a vortex phase mask (463 nm mask, vortex plate VPP-A, RPC Photonics, Rochester, NY). The first two foci were both generated by the same laser diode (50 mW, Calypso 50, Cobolt, Stockholm, Sweden). The third focal spot, again with a normal diffraction-limited Gaussian profile, was generated by a laser diode at 405 nm wavelength (30 mW, BCL-030-405-S, CrystaLaser, Reno, NV, USA) and used for the on-switching of rsEGFP2.

The microscope was equipped with a glycerol-immersion objective lens (PL APO, CORR CS, 63×, 1.3NA, glycerol; Leica Microsystems, Wetzlar, Germany). A piezo system (ENV40/20, Piezosystem Jena, Jena, Germany) was used to move the objective lens along the optical axis. A separate piezo stage (NV40, Piezosystem Jena) was implemented to translate the sample with nanometer precision in the xy-plane. The fluorescence signal was filtered by a band pass filter (532/70 nm) and detected by an avalanche photo diode (Perkin Elmer, Waltham, MA, USA); fluorescence photons were only allowed to be counted when the 491 nm read-out beam was switched on. The individual laser beam paths were triggered either by an acousto-optic modulator (MTS 130A3, Pegasus Optik GmbH, Wallenhorst, Germany) or by an acousto-optic tunable filter (AOTF.nC/TN, Pegasus Optik GmbH). The pulse sequence and duration were defined by a pulse generator (Model 9514, QUANTUM COMPOSERS, Bozeman, MT, USA) and triggered by a fast acquisition card (MCA-3 Series/P7882, FAST ComTec GmbH, Oberhaching, Germany) pixel by pixel.

Alternatively, we assembled the Abberior RESOLFT Quad P microscopy kit provided by Abberior Instruments GmbH, Göttingen, Germany, which used the same arrangement and wavelengths as the home-built system, except for the fact that scanning was accomplished by a galvanometer beam scanning system (Quad scanner) and the body of the microscope was an Olympus iX81 inverted microscope. Imaging was performed with a 100× Olympus oil immersion objective lens of 1.4 numerical aperture.

### Image acquisition and analysis

Image acquisition was performed with the software Imspector (www.imspector.de). Each image was recorded by applying a specific pulse scheme, pixel by pixel. The fluorescence signal was recorded only when the 491 nm read-out Gaussian shaped beam was on. Between each pixel pulse sequence (pixel dwell times 75–380 µs) a delay of 20 µs was inserted for synchronization, resulting in effective dwell times of 95–400 µs. The laser intensities used in our illumination scheme ranged between 1–100 kW/cm^2^. The approximately 10% remaining switching background introduces some diffraction-limited components in the final raw image. To remove this background, we deconvolved the final image by Richardson–Lucy (Richardson, 1972; Lucy, 1974) restoration with a 10% diffraction-limited PSF added to the RESOLFT PSF, as detailed previously (Hofmann et al., 2005). 10 iterations were performed. All experiments were performed at 35°C except those presented in Figure 3B and Figure 3—figure supplement 2.

### Determination of the single-molecule brightness

The single-molecule brightness of EGFP, rsEGFP and rsEGFP2 were determined using fluorescence fluctuation spectroscopy, specifically fluorescence correlation spectroscopy (FCS) (Haustein and Schwille, 2003) and fluorescence intensity distribution analysis (Chen et al., 1999; Kask et al., 1999). Both methods analyze characteristic fluctuations *δF*(*t*) in the fluorescence signal *F*(*t*) in time *t* about an average value *F*(*t*) = <*F*(*t*)> + *δF*(*t*) by either calculating the second-order auto-correlation function *G*(*t*_*c*_) (FCS, with correlation time *t*_*c*_) or by building up a frequency histogram *P*(*n*, ∆*T*) of photon counts detected per time window ∆*T* (FIDA, with number of photons *n*). Fluctuations in *F* arise for example from diffusion of the fluorescent proteins in and out of the confocal detection volume or by transitions into and out of a dark state such as the triplet, other metastable dark or the switch-off state.

FCS and FIDA data were analyzed using common theory. As outlined in detail previously (Eggeling et al., 2007), the analysis most importantly resulted in three characteristic molecular parameters of the fluorescent proteins: the single-molecule brightness (or count-rate per particle) *q* (from FIDA measurements with ∆*T* = 10 µs), the observation time *τ*_*obs*_ (from FCS measurements), and the average population of a µs-long-lived dark state (probably the triplet state of the fluorophore, from FCS measurements). In Figure 1—figure supplement 2 the *q* values in relation to the normalized *q* value of EGFP are shown. Without saturating the excitation, the brightness *q* ~ *Φ*_*FL*_ *ε* scales with the fluorescence quantum yield *Φ*_*FL*_ and the extinction coefficient *ε* (compare Table 1). For EGFP, the observation time *τ*_*obs*_ is given by its average transit time through the focal spot, while for rsEGFP and rsEGFP2 it is given by both the transit time and—if faster—the average switch-off time (Eggeling et al., 2007).

The fluorescence fluctuation data were recorded on a FCS reader (Insight, Inovation GmbH, Osnabrück, Germany), applying a water immersion objective (60× UPLSAPO, NA 1.2, Olympus, Japan). Data was recorded for different powers of the 491 nm excitation laser (Viper, Qioptiq, Hamble, UK) and for the fluorescent proteins in aqueous solution (PBS buffer, pH 7.5).

The observation times *τ*_*obs*_ were ~225 µs for EGFP (in accordance to the expected focal transit time), while those of rsEGFP and rsEGFP2 were shorter, reaching a value of ~40 µs at excitation powers >50 µW (15 kW/cm^2^) for rsEGFP and ~10–15 µs for rsEGFP2. The shorter observation times in the case of rsEGFP and rsEGFP2 result from a fast population of >200 µs-lived dark states (for details see (Eggeling et al., 2007)).
